# Decreased p53 is associated with a decline in asymmetric stem cell self‐renewal in aged human epidermis

**DOI:** 10.1111/acel.13310

**Published:** 2021-02-01

**Authors:** Alexandra Charruyer, Tracy Weisenberger, Hang Li, Ayman Khalifa, Andrew W. Schroeder, Annika Belzer, Ruby Ghadially

**Affiliations:** ^1^ Department of Dermatology UC San Francisco San Francisco California USA; ^2^ Department of Dermatology VA Medical Center San Francisco California USA; ^3^ Faculty of science Zagazig University Zagazig Egypt; ^4^ Department of Medicine UC San Francisco San Francisco California USA; ^5^ Yale School of Medicine New Haven Connecticut USA

**Keywords:** asymmetric division, epidermis, keratinocyte, p53, self‐renewal, stem cell

## Abstract

With age, the epidermis becomes hypoplastic and hypoproliferative. Hypoproliferation due to aging has been associated with decreased stem cell (SC) self‐renewal in multiple murine tissues. The fate of SC self‐renewal divisions can be asymmetric (one SC, one committed progenitor) or symmetric (two SCs). Increased asymmetric SC self‐renewal has been observed in inflammatory‐mediated hyperproliferation, while increased symmetric SC self‐renewal has been observed in cancers. We analyzed SC self‐renewal divisions in aging human epidermis to better understand the role of SCs in the hypoproliferation of aging. In human subjects, neonatal to 78 years, there was an age‐dependent decrease in epidermal basal layer divisions. The balance of SC self‐renewal shifted toward symmetric SC self‐renewal, with a decline in asymmetric SC self‐renewal. Asymmetric SC divisions maintain epidermal stratification, and this decrease may contribute to the hypoplasia of aging skin. P53 decreases in multiple tissues with age, and p53 has been shown to promote asymmetric SC self‐renewal. Fewer aged than adult ALDH+CD44+ keratinocyte SCs exhibited p53 expression and activity and Nutlin‐3 (a p53 activator) returned p53 activity as well as asymmetric SC self‐renewal divisions to adult levels. Nutlin‐3 increased Notch signaling (NICD, Hes1) and DAPT inhibition of Notch activation prevented Nutlin‐3 (p53)‐induced asymmetric SC self‐renewal divisions in aged keratinocytes. These studies indicate a role for p53 in the decreased asymmetric SC divisions with age and suggest that in aged keratinocytes, Notch is required for p53‐induced asymmetric SC divisions.

## Graphical abstract

Self‐renewal is decreased in aged human epidermis. Aging is associated with a shift in the balance of self‐renewal divisions toward increased symmetric and decreased asymmetric stem cell divisions. Fewer stem cells exhibit p53 expression and activity in aged versus young keratinocytes and p53 activation by Nutlin‐3 restores asymmetric stem cell self‐renewal divisions in aged keratinocytes.

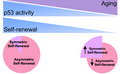

**Abbreviation**
SC: stem cell

## 1 INTRODUCTION

Aging is associated with a decline in integrity and function in most tissues. In epidermis, there is decreased stratification and differentiation. The average thickness of the epidermis is decreased (Kligman, 1979; Lavker, 1979; Lavker et al., 1987; Montagna & Carlisle, 1990), and there is retraction of the rete pegs (Giangreco et al., 2010). Epidermal maintenance requires a balance between self‐renewal and differentiation of SCs to ensure preservation of the SC population while simultaneously regenerating all suprabasal layers. It is believed that while relatively infrequent symmetric SC self‐renewal divisions maintain the SC pool (Morrison & Kimble, 2006), asymmetric SC divisions maintain epidermal stratification (Lechler & Fuchs, 2005). SC self‐renewal ability is decreased in aged murine tissues including muscle (Bernet et al., 2014; Cosgrove et al., 2014), brain (Moore et al., 2015), and blood (Florian et al., 2018). Similarly, self‐renewal ability in holoclone formation assay was decreased in aged murine hair follicle SCs (Keyes et al., 2013). Decreased holoclone formation was seen in two aged human keratinocyte samples (64y and 78y) compared with neonatal samples, further suggesting decreased self‐renewal related to age and/or development in humans (Barrandon & Green, 1987).

SC nomenclature can be inconsistent. Here, we rely on the definition of a SC as a cell that is uniquely able to self‐renew. Mammalian epidermal SCs are able to perform both symmetric and asymmetric SC self‐renewal divisions (Charruyer et al., 2017; Nöske et al., 2016; Poulson & Lechler, 2010). Asymmetric self‐renewal divisions lead to one SC and one committed progenitor and symmetric self‐renewal divisions lead to two SCs. SCs can also undergo symmetric differentiation divisions (leading to two committed progenitors). Committed progenitor/transit‐amplifying cells that result from SC divisions undergo 3–5 rounds of divisions before terminal differentiation, such that the majority of symmetric differentiation divisions constitute committed progenitor cell divisions.

In epidermis, divisions oriented perpendicular to the basement membrane are asymmetric SC self‐renewal divisions in both mouse (Blanpain & Fuchs, 2009; Lechler & Fuchs, 2005; Niessen et al., 2013; Poulson & Lechler, 2010; Williams et al., 2011) and human (Charruyer et al., 2017), generating a basal SC and an apical differentiated/committed progenitor. In human skin, symmetric divisions in the basal layer may represent symmetric differentiation (from SCs or differentiated progenitor cells), or symmetric self‐renewal divisions (from SCs). Suprabasal divisions are common in human skin and can both arise from and produce differentiated keratin 10 expressing committed progenitor/transit‐amplifying cells (Nöske et al., 2016) and therefore are considered divisions of differentiated (committed progenitor) cells. In summary, in the basal layer, asymmetric SC self‐renewal divisions arise from SCs, while symmetric divisions may arise from SCs or differentiated progenitor cells. In the suprabasal layers, divisions are differentiated progenitor, not SC divisions.

While studies in humans are few, multiple murine studies show a change in the proportion of symmetric/ asymmetric SC self‐renewal divisions with age. In muscle SCs from aged mice, the proportion of asymmetric divisions decreased significantly, to 60% from 80% (mice 23 vs. 4 months). In addition, in aged versus adult muscle SCs there was a decrease in the expression of genes associated with asymmetric SC self‐renewal (Bernet et al., 2014). In hematopoietic SCs from aged mice, the proportion of asymmetric SC divisions decreased to 35% from 80% (mice 20–26 vs. 3 months) (Florian et al., 2018). Restoring asymmetric divisions in aged hematopoietic SCs using a Cdc42 GTPase inhibitor rejuvenated the engraftment ability of SCs after transplantation (Florian et al., 2018). In summary, a decrease in asymmetric SC divisions appears to be a characteristic of multiple aged murine tissues.

Previous studies indicate that p53 promotes asymmetric SC self‐renewal divisions (Charruyer et al., 2017; Cicalese et al., 2009). While 80% of self‐renewal divisions in wild‐type murine mammary SCs were asymmetric, only 22% were asymmetric in mice with deactivated p53 (p53−/−). Also, reactivation of p53 in ErbB2 tumors (tumors in mice with attenuated p53 activity), using Nutlin‐3, restored asymmetric SC self‐renewal divisions and reduced tumor volume (Cicalese et al., 2009). Similarly, inhibition of p53 activity in normal human keratinocytes, using pifithrin alpha, decreased asymmetric SC divisions (Charruyer et al., 2017).

P53 expression and activity decline with age in neural and mesenchymal murine progenitors/SCs (Mikheev et al., 2009; Wilson et al., 2010). P53 activity, measured by target genes p21, MDM2, and cyclinG1, was decreased in aged murine epidermis (Feng et al., 2007). In human epidermis, p53 expression declined with age and was absent in subjects over 60 years old (Kim, Kang, et al., 2015).

There is a paucity of studies investigating alterations in SC self‐renewal in human aging. To address our hypothesis that SC self‐renewal is altered in aged human epidermis, we used immunofluorescence studies of SC divisions in human epidermal tissue sections and in keratinocytes in vitro. Given previous findings indicating that p53 promotes asymmetric SC self‐renewal, we hypothesized that the reduced p53 in aged human epidermis is associated with a decrease in asymmetric SC self‐renewal. P53 expression and activity were measured in aged versus adult keratinocyte SC populations, and Nutlin‐3 was used to assess the effect of p53 activation on asymmetric self‐renewal and differentiation in aged keratinocytes. Finally, an inhibitor of γ‐secretase was used to determine the requirement of Notch activation in p53‐induced asymmetric SC divisions.

## 2 EXPERIMENTAL PROCEDURES

### 2.1 Human skin samples

Human skin surgical discards were obtained with appropriate approvals from the UCSF Committee on Human Research. All studies abided by the rules of the Internal Review Board and the tenets of the Declaration of Helsinki. Samples were from aged subjects 70–92 years and adult subjects 25–50 years.

### 2.2 Perpendicular and parallel keratinocyte divisions in tissue sections

Neonatal, adult, and aged human skin samples were formalin‐fixed and paraffin‐embedded. Five to 8 micrometer sections were incubated with anti‐α tubulin (clone TU‐01,13‐8000, Invitrogen) and anti‐γtubulin (clone GTU‐88, ab11316, Abcam) (microtubules and centrioles, respectively) antibodies and AlexaFluor 488 secondary antibodies (Invitrogen), to identify spindle orientation of cell divisions. DAPI was used to identify nuclei. Fluorescence was detected using a Zeiss Axioplan 2 microscope or a confocal microscope (Nikon Eclipse Ti). The angle between the spindle axis and the basal layer (angle of division) was measured using the angle tool from ImageJ^®^ software. Divisions with angles less than 30° were considered parallel and divisions with angles between 60° and 90° were considered perpendicular (Charruyer et al., 2017; Lechler & Fuchs, 2005). Basal layer epidermal length was measured using ImageJ^®^.

### 2.3 Asymmetric and symmetric keratinocyte divisions in vitro

To analyze the first mitotic divisions in vitro, keratinocytes were isolated from freshly obtained adult and aged human skin samples using Dispase 25 U/ml, followed by 0.05% trypsin‐EDTA, and keratinocytes (unselected/unsorted) were plated in chamber slides with 154CF medium (0.07 mM calcium chloride) (Thermo Fisher Scientific). Twenty‐four hours later, cells were fixed with 3% paraformaldehyde, incubated with anti‐p53 (DO‐1, ab1101), anti‐Notch1 (ab4498b), anti‐Numb (ab4147), and anti‐keratin 1 (ab93652) primary antibodies (Abcam), followed by either AlexaFluor 488 or 568 secondary antibodies (Invitrogen). Divisions were identified as closely associated post‐mitotic sister pairs (forty to 100 divisions were analyzed for each sample). Greater than 98% of closely associated post‐mitotic sister pairs were BrdU+ at the employed seeding density, confirming cell division (Charruyer et al., 2016, 2017).

### 2.4 Nuclear expression of p53 and p21 in keratinocyte stem cells

Freshly obtained aged and adult skin samples were placed in dispase at the time of surgery and keratinocytes were isolated as described above. Then, an enriched population of ALDH+CD44+ keratinocyte SCs was sorted using a SONY SH800 FACS flow cytometer (Szabo et al., 2013a). ALDH^+^ cells were selected using ALDEFLUOR™ (Stem Cell Technologies) as previously described (Szabo et al., 2013a). CD44+ keratinocytes were selected using an allophycocyanin (APC)‐conjugated antibody (eBioscience). Isolated ALDH+CD44+ keratinocyte SCs were plated onto slides (no cell culture), fixed with 3% paraformaldehyde, permeabilized with 0.5% Triton X‐100, and incubated with anti‐p53 primary antibody (DO‐1, ab1101, Abcam) and anti‐p21 primary antibody (MA5‐14949, Invitrogen), followed by incubation with AlexaFluor 488 and AlexaFluor 568 secondary antibodies (Invitrogen), respectively. Mounting medium containing DAPI was used to identify nuclei. Numbers of cells with nuclear expression of p53 and/or p21 were quantified. We analyzed 100 ALDH+CD44+ keratinocyte SCs for each sample.

### 2.5 RNA sequencing

ALDH+CD44+ SCs and ALDH‐CD44‐ non‐SCs were collected from aged and adult human keratinocytes by flow cytometry, as described above. ALDH+CD44+ SCs and ALDH‐CD44‐ non‐SCs from aged and adult were placed in lysis buffer and Ambion RNAqueous^®^‐Micro Total RNA Isolation Kit used to extract RNA from 2000 keratinocytes, resulting in 260/280 readings >2, and an average of 4 ng/µl per 2,000 human keratinocytes sample (~20 µl). Dynabeads^®^ mRNA DIRECT™ MicroPurification Kit was then used to purify the extracted RNA before library preparation using the Ovation^®^ Single Cell RNA‐Seq System. This was followed by RNA sequencing using HiSeq. Thirty million paired‐end reads of cDNA fragments were aligned to the human reference genome. Reads were aligned to the human genome GRCh38 and quantified using the STAR aligner software version 2.7.2b. Differential expression analysis was performed in the R computing environment version 3.6.1 using the software DESeq2 version 1.26. For RNA sequencing, FDR‐corrected *p*‐values were used to evaluate significant differences between aged and adult keratinocyte SCs and non‐SCs using a significance threshold of 0.05. A minimum differential expression of log2 fold change ±1 was also used to determine significance. Pathway and gene ontology analyses were conducted by KEGG and DAVID, respectively.

### 2.6 Effect of p53 activation on asymmetric and symmetric stem cell self‐renewal

Freshly isolated keratinocytes from aged epidermis were incubated with Nutlin‐3 10 µM, DMSO (vehicle), N‐[N‐(3,5‐Difluorophenacetyl)‐L‐alanyl]‐S‐phenylglycine t‐butyl ester (DAPT, an inhibitor of γ‐secretase and Notch activation) 50 µM, or Nutlin‐3 plus DAPT (all from Sigma) for 48 h in chamber slides with 154CF medium. Keratinocytes were then fixed with 3% paraformaldehyde and incubated with anti‐Numb and anti‐keratin 1 primary antibodies, followed by AlexaFluor 488 and 568 secondary antibodies. Mounting medium with DAPI was used to identify nuclei. Forty to 100 divisions were analyzed for each sample.

### 2.7 Endoreplication

Tissue sections of aged and adult epidermis were stained with H&E and examined for presence of endoreplication figures (polyploid cells, indicating replication of the genome without subsequent cell division) (Zanet et al., 2005). As a second method, live cell imaging was used. Keratinocytes were isolated from freshly obtained aged and adult human skin samples and then placed in culture at 37°C with 5% CO_2_, in 154CF medium. Time lapse images were acquired every 20 min for 7 days to generate movies (Incucyte^®^ System). Culture media were replaced every 2–3 days. Movies generated were analyzed by manually tracking each cell and its’ subsequent divisions.

### 2.8 NICD, Hes1, keratin 1, keratin 10, and involucrin expression in Nutlin‐3‐treated aged keratinocytes

Freshly isolated keratinocytes from aged epidermis were incubated in 154CF medium supplemented with 10 µM Nutlin‐3 (Sigma) or vehicle, for 48 h. Keratinocytes were then fixed with 3% paraformaldehyde, permeabilized with 0.5% Triton X‐100, and then incubated with anti‐NICD (ab8925, Abcam), anti‐Hes1 (AF3317, R&D systems) (Notch pathway), anti‐keratin 1 (ab93652, Abcam), anti‐keratin10 (clone SPM262, SC‐56518, Santa Cruz Biotechnology), or anti‐involucrin (ab53112, Abcam) primary antibodies, followed by either AlexaFluor 488 or 568 secondary antibodies. Mounting medium with DAPI was used to identify nuclei. 500 cells were analyzed per *n*.

### 2.9 Statistical analysis

**FIGURE 1:**
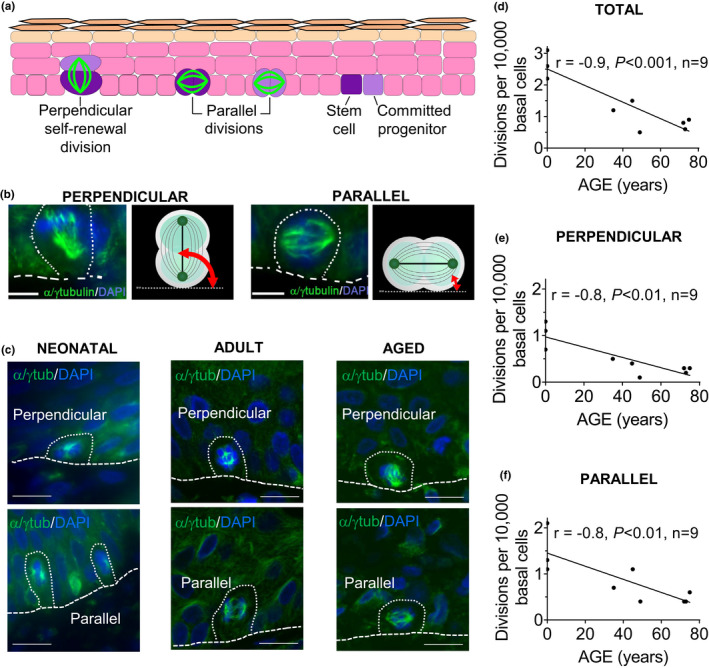
Total, perpendicular, and parallel divisions in the basal layer of human epidermis decrease in an age‐dependent manner. The number of divisions in the epidermal basal layer of human skin biopsies, from neonatal subjects to those 78 years old, was analyzed using immunofluorescence of tissue sections. (a) Illustration showing perpendicular (asymmetric) and parallel (symmetric SC self‐renewal, symmetric SC differentiation, or symmetric committed progenitor differentiation) divisions in epidermis. (b) Red arrow shows the method of measuring the angle between the spindle axis and the basal layer, using α/γ tubulin immunofluorescence. (c) Confocal images showing representative perpendicular and parallel divisions in neonatal, adult, and aged human epidermal basal layer. Scale bars 10 µm. (d, e, f) Correlation between numbers of divisions in the basal layer and age. Pearson correlation coefficient (*r*) between the number of (d) total divisions, (e) perpendicular (asymmetric SC self‐renewal) divisions, (f) parallel divisions per 10,000 basal cells, and age

**FIGURE 2:**
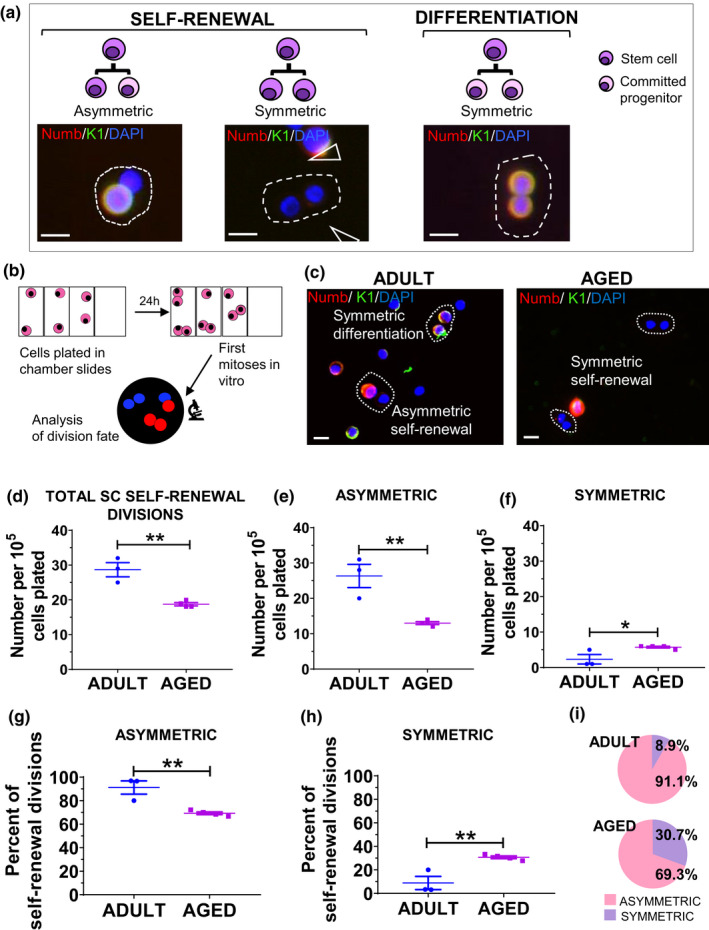
Asymmetric stem cell self‐renewal divisions are decreased, and symmetric stem cell self‐renewal divisions are increased, in aged versus adult human keratinocytes. (a) Illustration showing asymmetric and symmetric SC self‐renewal divisions, as well as a symmetric differentiation division in human keratinocytes. Scale bars 10 µm. (b) Methods used to analyze SC division fate of the first division after plating. (c) Immunofluorescence studies for keratin 1 and Numb expression. Representative asymmetric and symmetric SC self‐renewal divisions and symmetric differentiation division in human keratinocytes. Scale bars 10 µm. (d) The total number of SC self‐renewal divisions was decreased in aged versus adult keratinocytes. (e) The number of asymmetric SC self‐renewal divisions was decreased in aged versus adult keratinocytes. (f) The number of symmetric SC self‐renewal divisions was increased in aged versus adult keratinocytes. (g) The proportion of asymmetric SC self‐renewal divisions was decreased and (h) the proportion of symmetric SC self‐renewal divisions was increased in aged versus adult keratinocytes. (i) Pie charts showing proportions of asymmetric and symmetric SC self‐renewal divisions from g and h. Values are expressed as mean ± SEM and analyzed using a Student's *t* test. *p < 0.05, **p < 0.01

**FIGURE 3:**
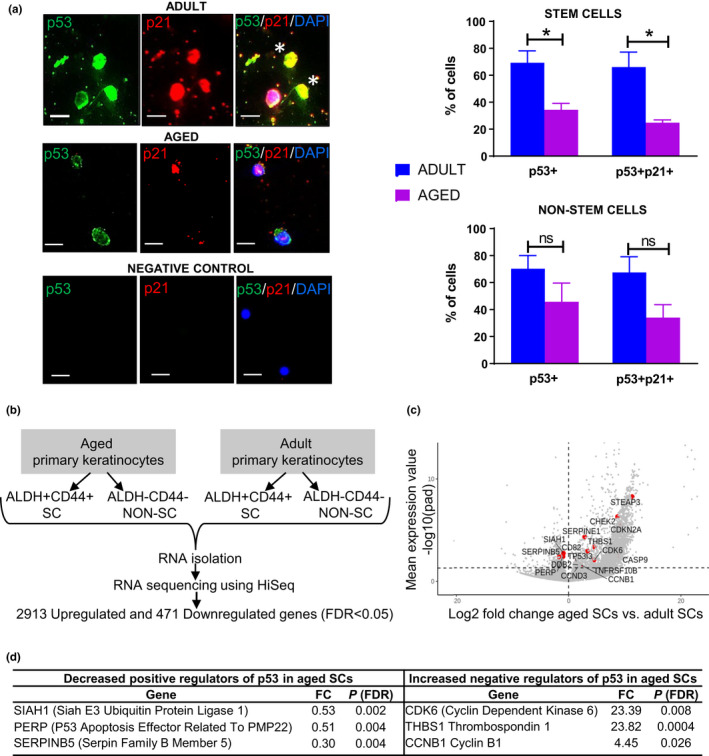
Aged ALDH+CD44+ keratinocyte stem cells exhibit decreased p53 expression and activity compared with adult. (a) Immunofluorescence study showing p53 nuclear expression and p21 nuclear expression (evidence for p53 activity) in adult and aged keratinocyte SCs. Asterisks indicate keratinocyte SCs positive for p53 and p21 nuclear expression. Negative control without primary antibody. Scale bars 10 µm. Upper histogram showing a significant decrease for ALDH+CD44+ SCs, in p53 and p53/p21 nuclear expression in aged versus adult (*n* = 3, separate individuals, 100 ALDH+CD44+ SCs per sample). Also, lower histogram showing a trend toward a decrease for ALDH−CD44− non‐SCs in p53 (*p* = 0.19) and p53/p21 (*p* = 0.06) nuclear expression in aged versus adult (*n* = 3, separate individuals, 100 ALDH−CD44− non‐SCs per sample). Values are expressed as mean ±SEM and analyzed using a Student's *t* test. *p < 0.05. (b) RNA sequencing of human aged versus adult SCs and non‐SCs. (c) Volcano plot displaying differentially expressed genes between aged SCs and adult SCs. The red dots represent the transcripts belonging to the p53 signaling pathway family. Positive x‐values represent up‐regulation and negative x‐values represent down‐regulation. (d) Underlying the decreased p53 signaling pathway (KEGG analysis) in aged versus adult SCs were positive regulators of the p53 pathway that show decreased expression with age and negative regulators that show increased expression with age

**FIGURE 4:**
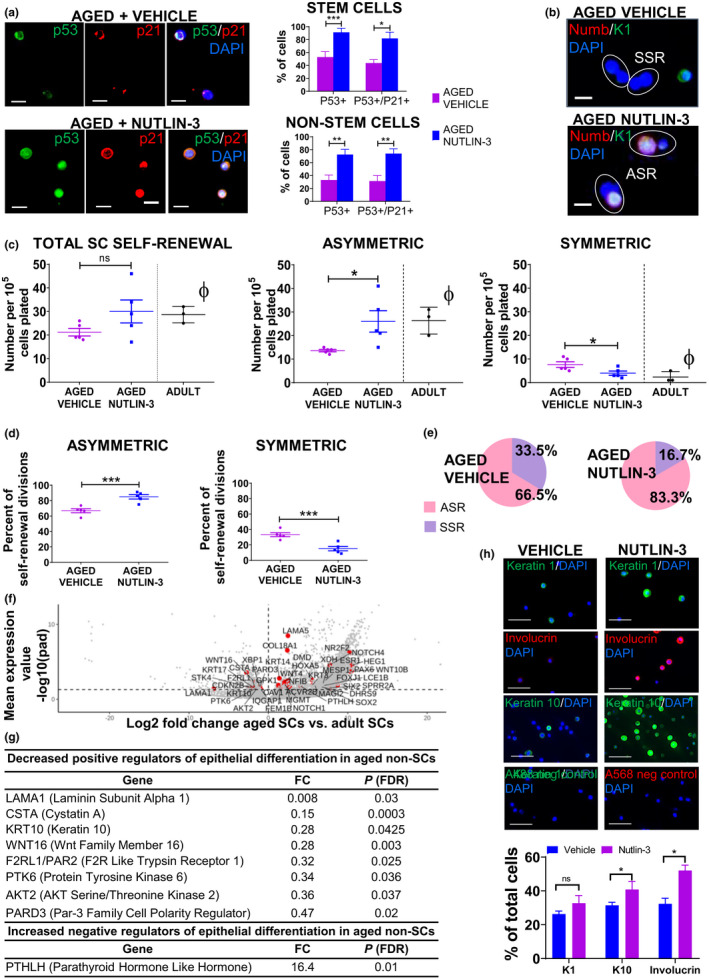
Nutlin‐3 activation of p53 in aged keratinocytes results in an increase in asymmetric stem cell self‐renewal divisions and increased expression of differentiation markers. (a) Immunofluorescence and histogram showing that Nutlin‐3 restores p53 expression and activity (p53+/p21+) in aged SCs and non‐SCs. (b) Representative immunofluorescence (keratin 1 and Numb) showing asymmetric and symmetric SC self‐renewal divisions in Nutlin‐3 versus vehicle‐treated aged human keratinocytes. Scale bars 10 µm. (c) Following Nutlin‐3 treatment in vitro, the total number of SC self‐renewal divisions is not significantly altered, the number of asymmetric SC self‐renewal divisions is significantly increased, and the number of symmetric SC self‐renewal divisions is significantly decreased. (ϕ) For reference: levels in adults were taken from the studies of Figure 2f. (d) Accordingly, the proportion of asymmetric SC self‐renewal divisions per total self‐renewal divisions is increased in Nutlin‐3 versus vehicle‐treated aged keratinocytes while the proportion of symmetric SC self‐renewal divisions per total self‐renewal divisions is decreased. (e) Pie charts showing proportions of asymmetric and symmetric SC self‐renewal. (f) RNA sequencing of aged versus adult ALDH−CD44− non‐SCs. Volcano plot displaying differentially expressed genes between aged and adult. The red dots represent the transcripts belonging to the epithelial differentiation signaling pathway family. Positive x‐values represent up‐regulation and negative x‐values represent down‐regulation. (g) Underlying the decreased epithelial differentiation pathway (GO‐BP analysis) seen in aged versus adult were decreased positive regulators and an increased negative regulator. (h). Immunofluorescence study of keratin 1, keratin 10, and involucrin expression following Nutlin‐3 versus vehicle treatment of aged keratinocytes. Histogram showing that Nutlin‐3 treatment increases the expression of differentiation markers (K1, K10 and Involucrin) in aged keratinocytes. Scale bars 50 µm. *p < 0.05, ***p < 0.001. Values are expressed as mean ± SEM and analyzed using a Student's *t* test. ASR, Asymmetric SC Self‐Renewal; SSR, Symmetric SC Self‐Renewal

**FIGURE 5:**
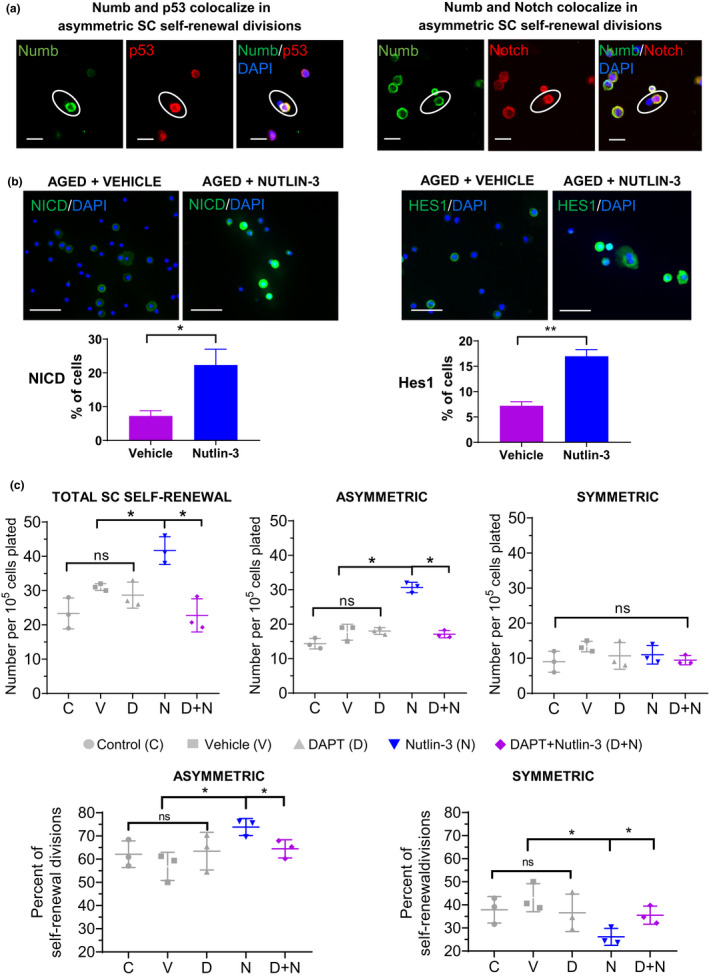
Notch activation is required for p53‐induced asymmetric SC self‐renewal divisions. (a) P53, Notch, and Numb immunofluorescence (*n* = 3 [separate human samples], for p53/Numb >90 divisions analyzed, for Notch/Numb >70 total divisions analyzed). Scale bars 10 µm. (b) Immunofluorescence study of nuclear NICD and Hes1 expression in Nutlin‐3 versus vehicle‐treated aged keratinocytes. Histograms showing the increased numbers of Nutlin‐3‐treated aged keratinocytes expressing nuclear NICD and Hes1 (*n* = 3, 500 cells analyzed per *n*). Scale bars 50 µm. (c) DAPT inhibition of Notch signaling in aged keratinocytes prevents the increase in both total SC self‐renewal and asymmetric SC self‐renewal divisions induced by Nutlin‐3 treatment. Symmetric SC self‐renewal divisions were unchanged for all treatment conditions. *p < 0.05, **p < 0.01. Values are expressed as mean ± SEM and analyzed using a one‐way repeated measures ANOVA

A correlation analysis was used for Figure 1d–f. A Student's *t* test was used to compare differences between means in Figure 2d–h, Figure 3a, Figure 4a–h, and Figure 5b. Repeated measures one‐way ANOVA was used for Figure 5c. Differences were considered significant if *p* ≤ 0.05.

## 3 RESULTS

### 3.1 Perpendicular and parallel divisions in the human epidermal basal layer decrease in an age‐dependent manner

The number of total divisions per 10 cm of the epidermal basal layer was studied using α/γ tubulin immunofluorescence in tissue sections of skin from human subjects, neonatal (day 0–4) to 78 years. α/γ tubulin immunofluorescence allowed us to detect divisions perpendicular to the basement membrane (asymmetric SC self‐renewal) and divisions parallel to the basement membrane (symmetric SC self‐renewal, symmetric SC differentiation, or committed progenitor symmetric differentiation), based on the division orientation. Both perpendicular divisions and parallel divisions were observed in neonatal, adult, and aged epidermis (Figure 1a–c). We determined the number of divisions per 10,000 basal cells. The total number of basal divisions per 10,000 basal cells decreased in an age‐dependent manner (Pearson correlation coefficient *r* = −0.9, *p* < 0.001, *n* = 9, Figure 1d), as did the number of perpendicular divisions (*r* = −0.8, *p* < 0.01, *n* = 9, Figure 1e), and the number of parallel divisions (*r* = −0.8, *p* < 0.01, *n* = 9, Figure 1f). Similarly, total, perpendicular, and parallel divisions were decreased per 10 cm basal layer (Figure S1a–c). We also found that the number of suprabasal divisions per 10 cm basal layer and 10,000 basal cells decreased in an age‐dependent manner (Figure S1d). Thus, there is a decrease in both perpendicular (asymmetric) and parallel (symmetric) divisions with age. Because perpendicular divisions constitute asymmetric SC self‐renewal divisions (Blanpain & Fuchs, 2009; Charruyer et al., 2017; Lechler & Fuchs, 2005; Niessen et al., 2013; Poulson & Lechler, 2010; Williams et al., 2011), these results indicate an age‐dependent decrease in total divisions and in asymmetric SC self‐renewal divisions. While parallel divisions are decreased, it is not possible to comment on symmetric SC self‐renewal divisions in vivo, as parallel divisions constitute both a small number of symmetric SC self‐renewal divisions and a large number of differentiation divisions (Charruyer et al., 2017).

### 3.2 Asymmetric stem cell self‐renewal divisions are decreased while symmetric stem cell self‐renewal divisions are increased, in aged versus adult human keratinocyte stem cells, in vitro

Because of the small number of divisions visualized in tissue sections and the lack of a reliable method to distinguish the subtypes of parallel divisions in human tissue sections at this time, we undertook the study of SC divisions in freshly isolated (unsorted) keratinocytes. The differentiated keratin 1‐positive cell of SC divisions co‐expressed Numb in 93.3 ± 4.5 to 100% of cases (Table S1), consistent with previous findings showing co‐expression of Numb with involucrin (Charruyer et al., 2017). Thus, in vitro, it is possible to identify and quantify the cell fate of keratinocyte divisions using expression of Numb and/or keratin 1 to identify differentiated daughter cells of divisions (Charruyer et al., 2017) (Figure 2a,b).

We also confirmed the accepted assertion that self‐renewal is a unique property of SCs in our model. ALDH+CD44+ human keratinocytes were selected as an enriched population of SCs that are functional long‐term repopulating cells, multipotent, self‐renewing, and label‐retaining cells (58%) (Szabo et al., 2013b) and Figure S2a. In the ALDH+CD44+ SC population, 38% of the divisions were self‐renewal divisions (28.7 ± 6.6% asymmetric SC self‐renewal, 9.4 ± 3.5% symmetric SC self‐renewal, 61.9 ± 8.3% symmetric differentiation), while in the ALDH−CD44− committed progenitor population (non‐SC keratinocytes that divide), 94.8 ± 2.7% of the divisions were symmetric differentiation divisions (Figure S2b). This study confirms that, unlike the SC population, the committed progenitor population performs almost exclusively symmetric differentiation divisions.

SC self‐renewal divisions were then quantified in freshly obtained and isolated keratinocytes from aged (71–77 years, *n* = 4) versus adult (26–49 years, *n* = 3), plated in 154CF medium for 24 h. There was a decrease in the number of total SC self‐renewal divisions (18.8 ± 0.5 vs. 28.7 ± 2.0, *p* = 0.002, Figure 2c,d). There was a decrease in asymmetric SC self‐renewal divisions (13.0 ± 0.4 vs. 26.3 ± 3.3, *p* = 0.004, Figure 2c,e) and an increase in symmetric SC self‐renewal divisions (5.8 ± 0.3 vs. 2.3 ± 1.3, *p* = 0.03, Figure 2c,f). Asymmetric SC self‐renewal constituted 91.1% of self‐renewal divisions in adults, consistent with our previous work (Charruyer et al., 2017). In aged versus adult keratinocytes, there was a decrease in the proportion of asymmetric SC self‐renewal divisions (69.3 ± 1.2% vs. 91.1 ± 5.6%, *p* = 0.006, Figure 2g,i and Figure S3) and a corresponding increase in the proportion of symmetric SC self‐renewal divisions (30.7 ± 1.2% vs. 8.9 ± 5.6%, *p* = 0.006, Figure 2h,i and Figure S3). We examined for differences in attachment between aged and adult keratinocytes. No significant difference in attachment was detected in aged versus adult (248.9 ± 58.5 vs. 245.4 ± 104.6 keratinocytes/mm^2^, *n* = 3, ns). We also examined for differences in the number of ALDH+CD44+ SCs between aged and young. No significant difference was detected in the number of ALDH+CD44+ in aged versus adult (1.2 ± 0.3% vs. 1.1 ± 0.2%, *n* = 5, ns, Figure S2c). In summary, asymmetric SC self‐renewal decreases with age, while symmetric SC self‐renewal increases, resulting in a shift in the balance of self‐renewal divisions toward symmetric SC self‐renewal. Additionally, the decrease in SC divisions with age appears to result from a decrease in SC activity (increase in quiescence) rather than a change in SC number.

### 3.3 P53 nuclear expression and activity are decreased in aged stem cells

P53 promotes asymmetric SC self‐renewal divisions (Charruyer et al., 2017; Cicalese et al., 2009; Tosoni et al., 2015), and p53 expression and activity were decreased in epidermis with age (Feng et al., 2007; Kim, Kang, et al., 2015). We examined the expression of p53 in aged (71–84 years) and adult (33–39 years) ALDH+CD44+ keratinocyte SC‐enriched populations (Charruyer et al., 2017; Szabo et al., 2013a).

P53 function depends on its nuclear localization (O’Brate & Giannakakou, 2003). In the aged, the number of ALDH+CD44+ keratinocyte SCs expressing nuclear p53 was decreased to approximately half of the adult (34.4 ± 4.7 vs. 69.3 ± 8.8, *p* = 0.01, *n* = 3, Figure 3a). No significant difference was detected in the number of ALDH−CD44− keratinocyte non‐SCs expressing nuclear p53 (45.8 ± 13.8% vs. 70.3 ± 9.7%, *p* = 0.19, *n* = 3, Figure 3a), although a downward trend was observed. P21, a p53 downstream target, was assessed as evidence for p53 activity. The number of ALDH+CD44+ keratinocyte SCs expressing both nuclear p53 and p21 was decreased in aged versus adult (24.8 ± 2.1% vs. 66.1 ± 11.1%, *p* = 0.01, *n* = 3, Figure 3a), as was the number of ALDH−CD44− keratinocyte non‐SCs expressing both nuclear p53 and p21 (34 ± 9.6% vs. 67.5 ± 11.6%, *p* = 0.06, *n* = 3, Figure 3a). These results indicate a decrease in p53 expression and activity in keratinocyte SCs of the aged epidermis.

To further investigate the decrease in p53 with aging, we analyzed transcriptomic data from aged versus adult keratinocytes (Figure 3b). Keratinocytes were isolated from patient samples and ALDH+CD44+ SCs as well as ALDH−CD44− non‐SCs obtained by flow sorting. The employed methods generated robust libraries with protein coding percent similar to the Universal Human Reference samples. Our samples expressed an array of keratinocyte‐related genes including epidermal stem/progenitor markers (e.g., K14, K17, LMO4, K15, and FGF18), genes linked to epidermal stratification or differentiation (K1, K10, KRTDAP, DMKN, S100A11, LOR, Involucrin, and FGFR2), and epidermal gap junctions, tight junctions or adhesion (GJB6, GJA1, COL18A1, COL17A1, LGALS7, LGALS3, EMP1, IQGAP1, 2 and 3, and PKP4). Various dermal markers were absent (e.g., Dermo‐1, Msx‐1, procollagen 1A, alpha smooth muscle actin) in our samples. The differentially expressed genes that were upregulated/downregulated in aged versus adult SCs and non‐SCs are shown in Table S2. Gene ontology (GO) analysis using KEGG (Ogata et al., 1999) of differentially expressed genes showed that the p53 pathway is downregulated in aged versus adult SCs (*p* = 0.06) Figure 3c and Tables S2 and S3. Among the downregulated genes for p53 in SCs were positive pathway regulators including SIAH1, PERP, and SERPINB5 and among the upregulated genes were negative pathway regulators including CDK6, THABS1, and CCNB1 (Figure 3d). In non‐SCs, significantly downregulated pathways by gene annotation analysis (Kegg, GO‐BP) did not include the p53 pathway (Table S4). Thus, there is a decrease in p53 pathway genes as well as a decrease in p53 protein expression and activity in aged SCs.

### 3.4 Nutlin‐3 activation of p53 in aged keratinocytes results in an increase in asymmetric stem cell self‐renewal divisions and increased expression of differentiation markers

We then investigated the effect of Nutlin‐3, an activator of p53, on SC self‐renewal divisions in aged keratinocytes (human subjects 75–92 years), in vitro. Nutlin‐3 activates p53 by inhibiting the interaction between p53 and MDM2 (inducer of p53 proteasomal degradation) (Vassilev et al., 2004). We first confirmed that Nutlin‐3 restores p53 protein expression and activity in aged keratinocyte SCs using immunofluorescence studies (Figure 4a). The number of ALDH+CD44+ SCs expressing nuclear p53 and p53/p21 was significantly increased in Nutlin‐3‐treated versus vehicle‐treated aged keratinocytes (p53: 91.3 ± 6.4% vs. 53 ± 8.5%, *p* = 0.04 and p53/p21: 81.9 ± 9.6% versus 43.7 ± 5.4%, *p* = 0.05, *n* = 3, Figure 4a). Also, the number of ALDH‐CD44‐ non‐SCs expressing nuclear p53 and p53/p21 was significantly increased in Nutlin‐3‐treated versus vehicle‐treated aged keratinocytes (p53: 72.5 ± 8% vs. 33.1 ± 7.7%, *p* = 0.02 and p53/p21: 74 ± 7.4% versus 31.5 ± 8.6%, *p* = 0.02, *n* = 3, Figure 4a). Thus, Nutlin‐3 restores p53 expression and activity in both aged SC and aged non‐SC keratinocytes.

Nutlin‐3‐treated aged keratinocytes showed a significantly increased number of asymmetric SC self‐renewal divisions versus vehicle‐treated aged keratinocytes (26 ± 5.1 vs. 13.6 ± 0.6, *p* = 0.048, *n* = 5, Figure 4b,c), while the number of symmetric SC self‐renewal divisions was decreased (4 ± 1 vs. 7.6 ± 1.4, *p* = 0.03, *n* = 5, Figure 4b,c). As a result, Nutlin‐3‐treated aged keratinocytes showed a significantly increased proportion of asymmetric SC self‐renewal divisions versus vehicle‐treated aged keratinocytes (83.3 ± 3.6% vs. 66.5 ± 3.8%, *p* = 0.0007, *n* = 5, Figure 4d,e and Figure S4a) and the proportion of symmetric SC self‐renewal divisions was significantly decreased (16.7 ± 3.6% vs. 33.5 ± 3.8%, *p* = 0.0007, *n* = 5, Figure 4d,e and Figure S4b). These results indicate that Nutlin‐3 activation of p53 can restore the numbers of asymmetric and symmetric SC self‐renewal divisions to levels close to that observed in adult epidermis, suggesting a p53‐dependent mechanism for asymmetric SC divisions in aged epidermis.

Aged epidermis exhibits decreased stratification and differentiation (Palazzo et al., 2015). Gene ontology (GO) analysis using the biological process (BP) method (Ashburner et al., 2000) of the differentially expressed genes in aged versus adult ALDH−CD44− non‐SC keratinocytes showed “epithelial differentiation” at the top of the significantly downregulated GO terms (Figure 4f,g and Table S2). Among the downregulated genes for differentiation were positive pathway regulators including LAMA1, CSTA, KRT10, WNT16, F2RL1/PAR2, PTK6, AKT2, and PARD3. Accordingly, we next determined whether activation of p53 by Nutlin‐3 would improve the altered differentiation phenotype seen in aging. Immunofluorescence studies of total (unsorted) aged keratinocytes from individual human skin samples (*n* = 3) showed that activation of p53 by Nutlin‐3 restored expression of proteins involved in early (keratin 1, keratin 10) and late (involucrin) differentiation (Figure 4h).

While our results show a role for asymmetric self‐renewal in the altered differentiation and hypoplasia of aged skin, endoreplication (cells undergoing replication of the genome in the absence of subsequent cell division) could also play a role. We first examined tissue sections of aged versus adult epidermis for polyploidy/endoreplication in the suprabasal epidermis using the method of Zanet et al., 2005. No significant difference was detected in the number of endoreplications (4.8 ± 1.6 vs. 5.8 ± 1 per mm of basal membrane) (Figure S5a). We then used live cell imaging of aged versus adult keratinocytes over 7 days, to determine the frequency of endoreplication, as evidenced by non‐resolving binucleation in growing colonies (Video 1, green box). No significant difference was detected in the number of endoreplications in aged versus adult keratinocyte colonies in vitro (25.7 ± 3.9% vs. 26.7 ± 3.9%) (Figure S5b). Thus, both methods indicate that there is not a significant role for changes in endoreplication in the altered differentiation and hypoplasia.

### 3.5 Notch signaling is required for Nutlin‐3‐induced asymmetric SC self‐renewal in aged keratinocytes

Because Numb controls p53 activity and p53 family members regulate Notch (Colaluca et al., 2008; Tosoni et al., 2015), we investigated the connection between p53, Notch signaling and asymmetric SC self‐renewal divisions in our in vitro model (Figure 5). P53 and numb were co‐segregated into the differentiated daughter cell in 87.9 ± 4.2% of asymmetric SC self‐renewal divisions in human keratinocytes (*n* = 3, separate human samples, Figure 5a, left panels). Notch and numb were co‐segregated into the differentiated daughter cell in 93.8 ± 4.2% of asymmetric SC self‐renewal divisions in human keratinocytes (*n* = 3, separate human samples, Figure 5a, right panels). Thus, numb, notch, and p53 are all segregated to the differentiated daughter cell during asymmetric SC self‐renewal division.

We next determined whether Nutlin‐3 activates the Notch signaling pathway in aged keratinocytes. After Notch activation, Notch receptor is cleaved by γ‐secretase and the resulting Notch intracellular domain (NICD) is released. NICD migrates to the nucleus enabling transcription of Notch target genes, including Hes1 (Artavanis‐Tsakonas et al., 1995; Lai, 2002; Mumm & Kopan, 2000). Nuclear expression of NICD and Hes1 was quantified in unselected aged keratinocytes treated with Nutlin‐3 10 μM or vehicle, using immunofluorescence. Following Nutlin‐3 versus vehicle treatment, the number of aged keratinocytes expressing nuclear NICD (22.3 ± 4.7% vs. 7.3 ± 1.5%, *n* = 3, *p* = 0.05) and Hes1 (17 ± 1.3% vs. 7.2 ± 0.8%, *n* = 3, *p* = 0.002) was increased (Figure 5b).

We then investigated whether Notch signaling is required for Nutlin‐3 induced asymmetric SC self‐renewal divisions in aged keratinocytes. N‐[N‐(3,5‐Difluorophenacetyl)‐L‐alanyl]‐S‐phenylglycine t‐butyl ester (DAPT, inhibitor of γ‐secretase) inhibits Notch signaling by preventing cleavage of Notch receptor (Palazzo et al., 2015). Unselected aged keratinocytes were treated with Nutlin‐3, a combination of DAPT and Nutlin‐3, vehicle, DAPT alone, or not treated. Inhibition of Notch signaling in aged keratinocytes using DAPT prevented the Nutlin‐3‐induced increase in asymmetric SC self‐renewal divisions (16.9 ± 2.9 vs. 30.7 ± 0.9 per 100,000 plated cells) and the increase in total SC self‐renewal (21.7 ± 5.2 vs. 41.7 ± 2.3 per 100,000 plated cells) (Figure 5c and Figure S6). No significant difference in the number of symmetric SC self‐renewal divisions could be detected between any of the conditions (Figure 5c). Among the experimental controls, no significant difference could be detected in the number of total SC self‐renewal divisions or in the number of asymmetric SC self‐renewal divisions (Figure 5c). These data suggest that Notch signaling is required for the p53‐induced asymmetric SC self‐renewal in aged keratinocytes.

## 4 DISCUSSION

Our studies aimed to provide insight into changes in epidermal SC self‐renewal with aging, in humans. The majority of studies of SC self‐renewal and aging have been conducted in mice due to the availability of genetic models and murine tissues. Studies of SC self‐renewal in human skin are challenging, due to heterogeneity of skin from different body sites and increasing heterogeneity of human subjects with age, potentially leading to large standard deviations in data obtained. Our present study provides evidence that in human tissue, while SC self‐renewal divisions decrease with age, the asymmetric/symmetric balance shifts to favor symmetric self‐renewal. Treatment of aged keratinocytes with a p53 activator, Nutlin‐3, increased asymmetric SC self‐renewal divisions to levels similar to those in normal adults. In this study, Notch activation was required for the p53‐mediated increase in asymmetric SC self‐renewal divisions.

### 4.1 Stem cell self‐renewal decreases with age

In the presented studies, in vivo, both perpendicular and parallel divisions decreased with age and in vitro, total self‐renewal divisions decreased, consistent with previous results in mice (Bernet et al., 2014; Cosgrove et al., 2014; Florian et al., 2018; Keyes et al., 2013; Moore et al., 2015). The decrease in total self‐renewal divisions observed could reflect a decrease in total SC number or an increase in SC quiescence (decrease in SC activation). SC number does not change with age in mice (Charruyer et al., 2009; Giangreco et al., 2008), or in humans as evidenced by the unchanged number of ALDH+CD44+ keratinocytes with age (Figure S2c). Therefore, it appears that there is a decrease in SC activation (increase in quiescence), rather than a decrease in SC number with age. Studies of SC quiescence will be required to confirm this.

Many methods are used to investigate SC self‐renewal, including holoclone formation assays, lineage tracing, sphere‐forming assays, organoids, and organ reconstitution. However, imaging of division orientation and assessment of the partitioning of cell fate determinants in isolated cells using sister pair analysis in vitro are considered optimal methods for studying asymmetric versus symmetric self‐renewal (Santoro et al., 2016), providing a more granular assessment. Our in vitro studies provide larger numbers of divisions for analysis than in vivo. In vitro, we can distinguish symmetric SC self‐renewal from symmetric differentiation divisions (SC and committed progenitor) using keratin 1 and Numb, not possible at present in tissue sections. In vitro, it was possible to discern an increase in the absolute number of symmetric SC self‐renewal divisions, even in the face of an overall decline in total parallel/symmetric divisions with age (Figure 1f). Thus, while in vivo tissue sections provide in situ information, sister pair analysis in vitro is invaluable as a complementary method to characterize SC divisions.

### 4.2 Decreased asymmetric stem cell self‐renewal and aging

With aging, a decreased rate of asymmetric SC self‐renewal and an increased rate of symmetric SC self‐renewal divisions have been predicted, using mathematical modeling (Lynch, 2004). The decreased rate of asymmetric SC self‐renewal divisions would reduce the production of differentiated cells, resulting in a diminished ability of aged tissues to maintain homeostasis (Lynch, 2004). Later studies have supported this prediction. Muscle SCs from aged mice show a 50% decrease in asymmetric (phosphor‐p38) divisions, as well as a reduction in the expression of genes associated with asymmetric divisions (Bernet et al., 2014). Young murine hematopoietic SCs divide predominantly asymmetrically, while aged hematopoietic SCs have altered Cdc42 activity and divide predominantly symmetrically (Florian et al., 2018). A Cdc42‐specific inhibitor restored the frequency of asymmetric SC divisions in aged hematopoietic SCs and resulted in rejuvenation of SC tissue maintenance with improved contribution to B, T, and myeloid cell lineages after transplantation (Florian et al., 2018). While the above data were obtained from aged mice, in aged human keratinocytes manipulation of SC divisions with Phycosaccharide AI resulted in increased asymmetric SC self‐renewal divisions and improved SC holoclone formation ability (Charruyer et al., 2016). The present findings of decreased asymmetric SC self‐renewal divisions in aged epidermis indicate that decreased asymmetric SC self‐renewal is a feature of aging, not only in mice, but in humans.

### 4.3 Alterations in asymmetric stem cell self‐renewal divisions produce alterations in epidermal stratification with resultant skin pathology

In the epidermis, asymmetric SC self‐renewal divisions maintain the SC pool by replacement of the dividing SCs, while simultaneously replenishing epidermal layers through production of differentiated progenitor cells. Asymmetric SC self‐renewal divisions require the formation of an apical complex composed of polarity proteins. Previous experimental studies investigated the effects of deleting key polarity proteins on asymmetric SC self‐renewal divisions and epidermal stratification. After deletion of LGN (Leu‐Gly‐Asn repeat) in murine epidermis, asymmetric SC divisions were reduced, resulting in fewer suprabasal cells and a thinner epidermis (Williams et al., 2011). In a PDK1 (Phosphoinositide‐dependent kinase‐1) conditional knock out mouse, asymmetric SC self‐renewal divisions were decreased to 13% from 50% (wild type) and epidermal thickness was decreased (Dainichi et al., 2016). Thus, genetic manipulations in mice indicate that a decrease in asymmetric SC self‐renewal is associated with epidermal thinning.

**FIGURE 6:**
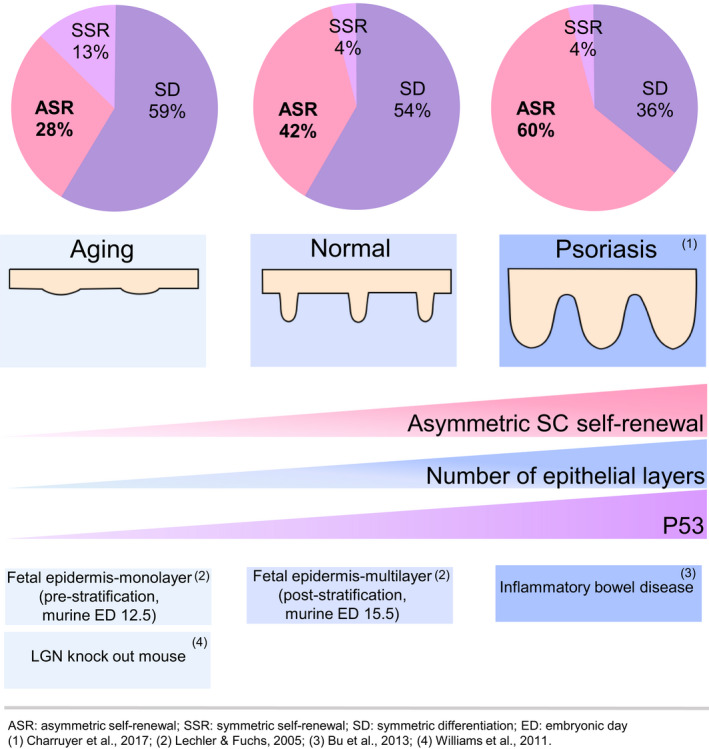
Asymmetric SC self‐renewal promotes epithelial stratification in development, disease, and aging. ASR, asymmetric self‐renewal, SSR, symmetric self‐renewal, SD, symmetric differentiation, ED, embryonic day

In both development and disease, changes in the balance of asymmetric and symmetric SC self‐renewal divisions occur (Figure 6). During murine embryonic growth, symmetric divisions of SCs increase the surface area of the single‐layered epidermis (Lechler & Fuchs, 2005). Later in development, asymmetric SC divisions become predominant, resulting in formation of the multi‐layered epidermis (Lechler & Fuchs, 2005; Smart, 1970). In human psoriasis, increased asymmetric SC self‐renewal divisions are associated with a profound increase in epidermal thickness (2–5 fold) (Kim, Nadella et al., 2015). The increase in asymmetric SC self‐renewal divisions in this hyperproliferative inflammatory skin disease was found to be IL17A‐dependent (Charruyer et al., 2017). Similarly, increased asymmetric SC self‐renewal divisions were seen in hyperproliferative inflammatory bowel disease in mice, induced by dextran sodium sulfate or TNFα (Bu et al., 2016). In addition, increased asymmetric SC self‐renewal divisions were thought to be the mechanism by which IL22 increases intestinal committed progenitors without affecting the number of intestinal SCs (Zwarycz et al., 2019). Thus, increased asymmetric SC self‐renewal divisions can result in production of excess differentiated cells and, in the case of epidermis, increased epidermal thickness, while decreased asymmetric SC self‐renewal divisions with age result in epidermal thinning, supporting the contention that asymmetric SC self‐renewal is responsible for epidermal stratification in development and disease.

Given that aging is associated with decreased asymmetric SC self‐renewal, aged epidermis might be expected to exhibit decreased stratification and differentiation. Consequences of decreased asymmetric SC self‐renewal include a decreased production of committed progenitor cells. The reduced population of committed progenitor cells undergoing symmetric divisions will result in fewer differentiated cells, thus contributing to hypoplasia. Average human epidermal thickness/stratification is decreased with aging (Kligman, 1979; Lavker, 1979; Lavker et al., 1987; Montagna & Carlisle, 1990). The epidermal differentiation marker K10 is decreased with age (Palazzo et al., 2015), and our RNA sequencing shows a decrease in epithelial differentiation genes with aging. Given that increasing asymmetric SC self‐renewal divisions in the aged (by activation of p53) increased differentiation, these findings suggest that the changes in asymmetric SC self‐renewal with aging have consequences for differentiation and stratification of the epidermis.

### 4.4 Role of p53 activation in stem cell self‐renewal

Mice lacking p53 show increased hematopoietic SC numbers but decreased repopulation ability, suggesting that the lack of p53 increases SC number but impairs SC functional self‐renewal ability (Chen et al., 2008; Liu et al., 2009; Akala et al., 2008; TeKippe et al., 2003; Pant et al., 2012). In p53 null mice, there were increased neural SCs capable of forming secondary spheres, indicating that lack of p53 increases SC number and promotes SC self‐renewal (Liu et al., 2013; Meletis et al., 2006). These contrasting observations may be a result of different methods used to assess self‐renewal or differences between neural and hematopoietic SCs. Similar to findings in both neural and hematopoietic tissues, our previous studies found an increase in SC number with p53 inhibition (Charruyer et al., 2017). Similar to studies in hematopoiesis and in contrast to studies in neurons, the present studies showed a decrease in SC self‐renewal (in both tissue sections and in vitro) with decreased p53 related to age.

Reports from epithelial tissues indicate that p53 plays a role in the regulation of the balance between asymmetric and symmetric SC self‐renewal divisions. Mammary SCs from p53 null mice underwent decreased asymmetric SC self‐renewal divisions, and treatment with the p53 activator Nutlin‐3 restored asymmetric SC divisions (Cicalese et al., 2009; Tosoni et al., 2015). Likewise, we previously showed that p53 inhibition, using pifithrin α, decreased keratinocyte asymmetric SC self‐renewal divisions (Charruyer et al., 2017). As expected, based on Cicalese et al. and Charruyer et al., the present work found that activating p53 in aged keratinocytes increased the numbers of asymmetric, and decreased the numbers of symmetric SC self‐renewal divisions. This resulted in restoration of the proportions of asymmetric and symmetric SC self‐renewal divisions to that similar to proportions in normal adults. Altogether, these data suggest a role for p53 in the switch between symmetric and asymmetric SC self‐renewal, not only in murine tissues but also in human epidermis.

While Nutlin‐3 increases asymmetric SC self‐renewal divisions, it is possible that other effects of increased p53 play a role in the improved differentiation. P53 has a role in apoptosis, such that activation of p53 in aged keratinocytes might induce apoptosis. However, there was increased cell proliferation with no increase in apoptosis in aged murine muscle SCs (Liu et al., 2018) and in mammary SCs (Tosoni et al., 2015) treated with Nutlin‐3 10 μM. Based on these reports, it is not expected that Nutlin‐3 would increase apoptosis in aged keratinocytes at the dose we use in our model (10 μM).

### 4.5 Role of the Notch pathway in p53‐induced asymmetric SC self‐renewal

The molecular mechanisms underlying the effects of p53 on asymmetric SC self‐renewal are not fully understood. Numb promotes p53 activity by binding to MDM2 and preventing p53 degradation (Colaluca et al., 2008; Tosoni et al., 2015). In mammary epithelial SCs, Numb and p53 are co‐segregated into the differentiated cell of asymmetric SC self‐renewal divisions. Mammary epithelial SCs from Numb knockout mice showed lower levels of p53 activity and failed to asymmetrically segregate p53 during divisions, leading to decreased asymmetric and increased symmetric SC divisions suggesting that Numb is a key regulator of p53 and SC self‐renewal (Tosoni et al., 2015). In keeping with these studies, we found that p53 is co‐segregated with Numb to the differentiated cell of asymmetric SC self‐renewal divisions and p53 activation is associated with increased asymmetric SC self‐renewal.

Williams et al reported that Notch signaling is a major downstream effector of the asymmetric SC self‐renewal division machinery and epidermal stratification (Williams et al., 2011). P53 positively regulated Notch1 receptor in human keratinocytes and inhibition of Notch resulted in lesser stratification in organotypic culture (Lefort et al., 2007). Rando et al also found a direct positive correlation (*R* ^2^ = 0.9) between p53 activity and Notch activity in aged muscle SCs (Liu et al., 2018). Notch1R and activated Notch (NICD) were reported to be decreased with age in keratinocyte SCs (Palazzo et al., 2015). In keeping with this, we found Notch is required for p53‐induced asymmetric SC self‐renewal divisions in aged skin.

## 5 CONCLUSIONS

Our findings indicate that while total cell divisions and specifically total SC self‐renewal divisions decrease with age, the balance of asymmetric/symmetric SC self‐renewal shifts to favor symmetric SC self‐renewal. Further, in aged epidermis, our studies suggest that decreased asymmetric SC self‐renewal contributes to the hypoplasia observed and one can speculate that the increased symmetric SC self‐renewal plays a role in skin cancer proclivity. Restoring p53 activity in aged keratinocytes promotes asymmetric SC division and normalizes the balance of SC division fate. Finally, this work supports the idea that manipulation of SC self‐renewal is an attractive strategy in the treatment of conditions involving epidermal hypo‐ and hyperproliferation, such as aging, wound healing, psoriasis, cancer, and hyperproliferative ichthyoses.

## CONFLICT OF INTEREST

The authors report no conflict of interest relevant to this article.

## AUTHOR CONTRIBUTIONS

AC and RG designed the research. TW, HL, AC, and AK performed experiments. AC, AB, AWS, and RG analyzed the data. RG provided samples. AC, AB, AWS, and RG wrote the manuscript.

### Open Research Badges

This article has been awarded <Open Data, Open Materials> Badges. All materials and data are publicly accessible via the Open Science Framework at https://doi.org/10.6084/m9.figshare.

## Supporting information

Supplementary Material

Table S2

Video S1

## Data Availability

The data that support the findings of this study are openly available in [Figshare] at https://doi.org/10.6084/m9.figshare.9253151 (Charruyer et al., 2019).
