# Supplementary material for: Decreased p53 is associated with a decline in asymmetric stem cell self‐renewal in aged human epidermis
Source: Aging Cell. 2021 Feb 1;20(2):e13310. doi: 10.1111/acel.13310 (PMC7884041; doi:10.1111/acel.13310)
Supplement: Supplementary file 1 — Supplementary Material [file ACEL-20-e13310-s001.pdf]

Supplementary Table 1

Sample descriptions	Numb/K1 correlation in asymmetric self-renewal divisions	Numb/K1 correlation in symmetric self-renewal divisions	Numb/K1 correlation in symmetric differentiation divisions
73 years (49 divisions)	100%	100%	100%
81 years (54 divisions)	100%	100%	100%
71 years (42 divisions)	100%	100%	95.8%
77 years (62 divisions)	77.3%	100%	100%
75 years (46 divisions)	89.5%	100%	95.2%
**Mean ± SEM**	**93.3 ± 4.5%**	**100 ± 0%**	**98.2 ± 1.1%**

Supplementary Table 1. Correlation of Numb and Keratin 1 expression in asymmetric and symmetric divisions. Immunofluorescence microscopy was performed on isolated keratinocytes plated for 24 hours to assess the first divisions in vitro. The differentiated keratin 1 positive cell of asymmetric SC self-renewal divisions co-expressed with Numb in 93.3±4.5% of cases. Both differentiated cells of symmetric differentiation divisions (keratin 1+) co-expressed Numb in 98.2±1.1% of cases. Both undifferentiated cells (keratin 1 -) of symmetric SC self-renewal divisions lacked Numb expression in 100±0% of cases.

Supplementary Table 3

Pathway analysis for aged versus adult SCs

**KEGG downregulated pathways**	*P* value	Number of genes
Oxidative phosphorylation	0.01	12
Protein processing in endoplasmic reticulum	0.02	14
mRNA surveillance pathway	0.05	10
P53 signaling pathway	0.06	16
**KEGG upregulated pathways**		
Peroxisome	0.04	14
PPAR signaling pathway	0.05	15
**GO-BP downregulated**		
Protein modification by small protein conjugation or removal	0.0007	79
Protein modification by small protein conjugation	0.0007	75
Catabolic process	0.001	231
Intracellular transport	0.001	156
Protein ubiquitination	0.001	72
**GO-BP upregulated**		
Nerve development	0.03	10
Positive regulation of cell division	0.04	12
Tumor necrosis factor superfamily cytokine production	0.04	11
Regulation of cell division	0.04	17
Cell recognition	0.04	10

Supplementary Table 4

Pathway analysis for aged versus adult non-SCs

**KEGG downregulated pathways**	*P*	Number of genes
Phagosome	0.006	20
Ubiquitin mediated proteolysis	0.04	15
Cell adhesion molecules (CAMs)	0.04	14
Endocytosis	0.05	35
**KEGG upregulated pathways**		
Protein digestion and absorption	0.02	11
Calcium signaling pathway	0.06	22
**GO-BP downregulated**		
Lipid biosynthetic process	0.0004	71
Leukocyte activation	0.0004	48
Cell activation	0.0007	78
Cellular lipid metabolic process	0.0009	128
Lymphocyte activation	0.001	42
Epithelial cell differentiation	0.007	43
**GO-BP upregulated**		
Appendage development	0.01	20
Limb development	0.01	20
Adult behavior	0.02	13
Embryonic skeletal system development	0.04	18
Heterocycle biosynthetic process	0.04	474

Supplementary Figure 1

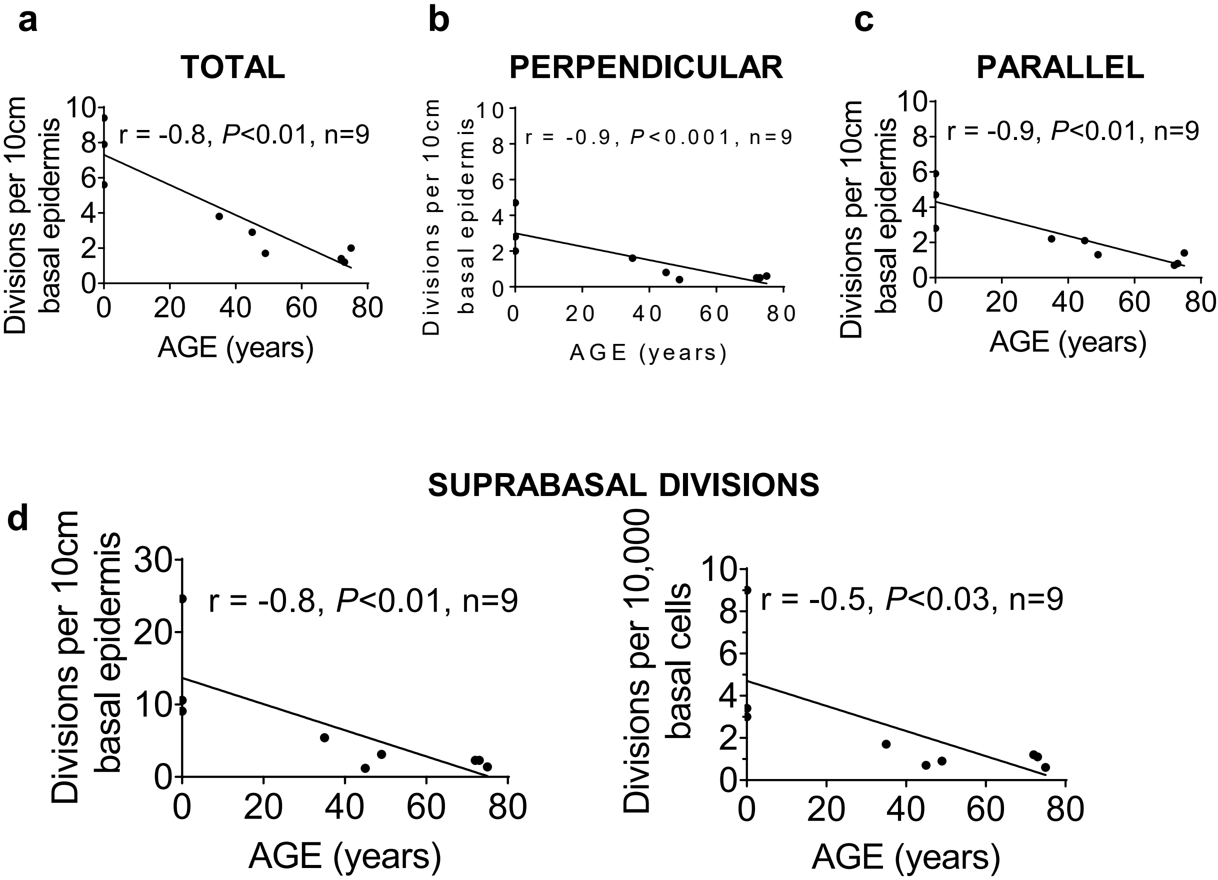

Supplementary Figure 1. The number of basal and suprabasal mitoses decreases in an age dependent manner (in support of Figure 1). The number of divisions in the epidermal basal and suprabasal layers of human skin biopsies, from neonatal subjects to those 78 years old, was analyzed using immunofluorescence of α/γ tubulin in tissue sections. (a) The total number of basal divisions per 10 cm basal layer decreased in an age-dependent manner (Pearson correlation coefficient $r=-0.8$, $P<0.01$, $n=9$). (b) The number of perpendicular divisions per 10 cm of basal layer decreased in an age-dependent manner ($r = -0.9$, $P<0.001$, $n=9$), as did (c) the number of parallel divisions ($r=-0.9$, $P<0.01$, $n=9$). (d) We also found that the number of suprabasal divisions per 10 cm basal layer decreased in an age dependent manner ($r=-0.8$, $P<0.01$, $n=9$). For the number of suprabasal divisions per 10,000 basal cells there was a moderate correlation with age ($r=-0.5$, $P<0.03$, $n=9$).

Supplementary Figure 2

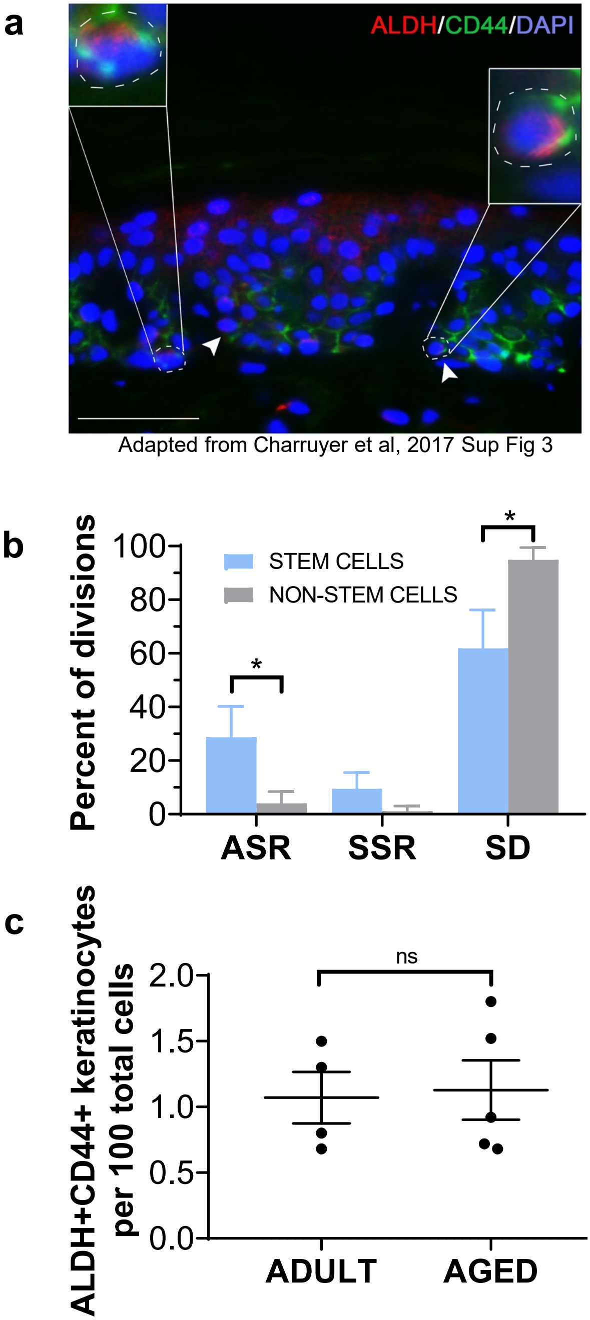

Supplementary Figure 2. ALDH+CD44+ epidermal SCs. (a) Immunofluorescence image of ALDH+CD44+ SCs in normal adult human epidermis. Anti-CD44 antibody (cell membrane) from Biolegend. Anti-ALDH1 antibody (cytoplasmic) from Sigma (Szabo et al 2013). It should be noted that this is ALDH protein expression rather than enzyme activity. (b) Asymmetric SC self-renewal divisions are primarily ALDH+CD44+ SC divisions versus ALDH-CD44- committed progenitor divisions. (c) No significant change was detected in the number of ALDH+CD44+ keratinocytes in aged (n=5) versus adult (n=4) human keratinocytes.

Supplementary Figure 3

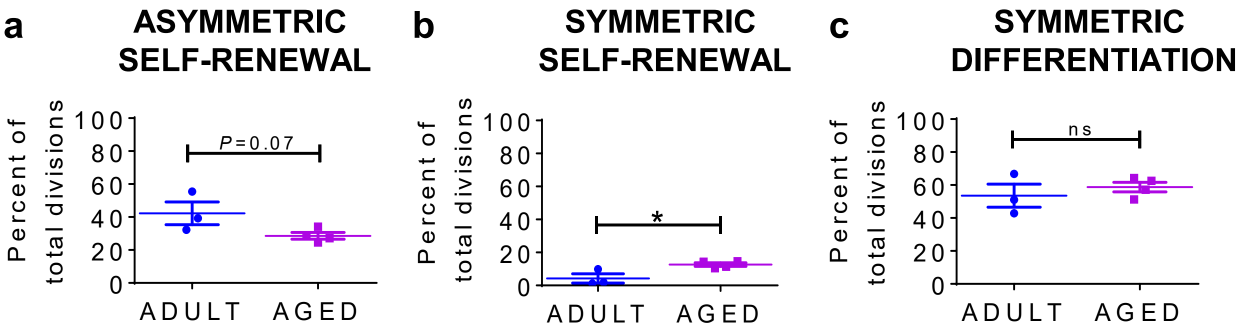

Supplementary Figure 3. The proportion of asymmetric and symmetric divisions per total cell divisions in aged (n=4) versus adult (n=3) keratinocytes (in support of Figure 2). (a) Asymmetric self-renewal divisions. (b) Symmetric self-renewal divisions. (c) Symmetric differentiation divisions (SC differentiation and committed progenitor differentiation divisions).

Supplementary Figure 4

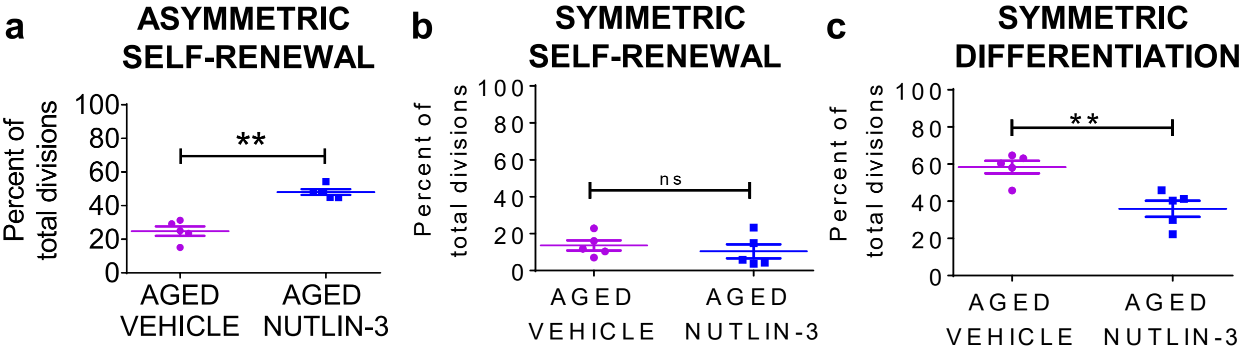

Supplementary Figure 4. The proportions of asymmetric and symmetric divisions per total cell divisions in Nutlin-3-treated (n=5) versus vehicle-treated (n=5) keratinocytes (in support of Figure 4). Proportion of (a) Asymmetric SC self-renewal divisions (b) Symmetric SC self-renewal (c) Symmetric differentiation divisions (SC differentiation and committed progenitor differentiation divisions).

Supplementary Figure 5

a

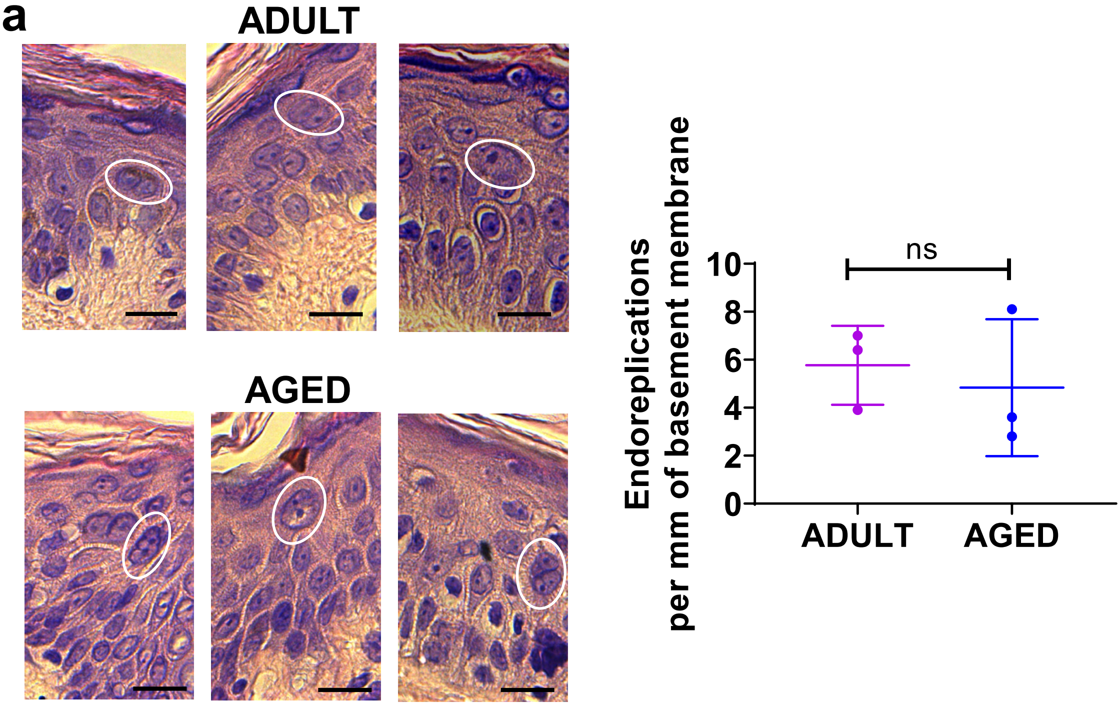

b

	ADULT (n=91)	AGED (n=185)	*P*
Number of colonies with at least one endoreplication /100 colonies	26.7 ± 3.9%	25.7 ± 8.3%	ns
Number of endoreplications /100 colonies	37.8 ± 9.4%	30.1 ± 11.8%	ns

Supplementary Figure 5. No significant difference was detected in the number of endoreplications in aged vs. adult epidermis. (a) H&E was used to determine the number of endoreplications in human epidermis from aged versus adult, in tissue sections. Results are expressed as the number of endoreplications per mm basal layer ± SEM (n=3). Scale bar = 10µm. (b) Time lapse microscopy was used to determine the number of endoreplications in colonies produced by aged versus adult keratinocytes. The number of endoreplications in a total of 185 aged versus 91 adult colonies over 7 days was examined (3 aged versus 3 adult individuals). The number of colonies with at least one endoreplication was determined and, because some colonies show multiple endoreplications, the number of endoreplications per 100 colonies was also determined.

Supplementary Figure 6

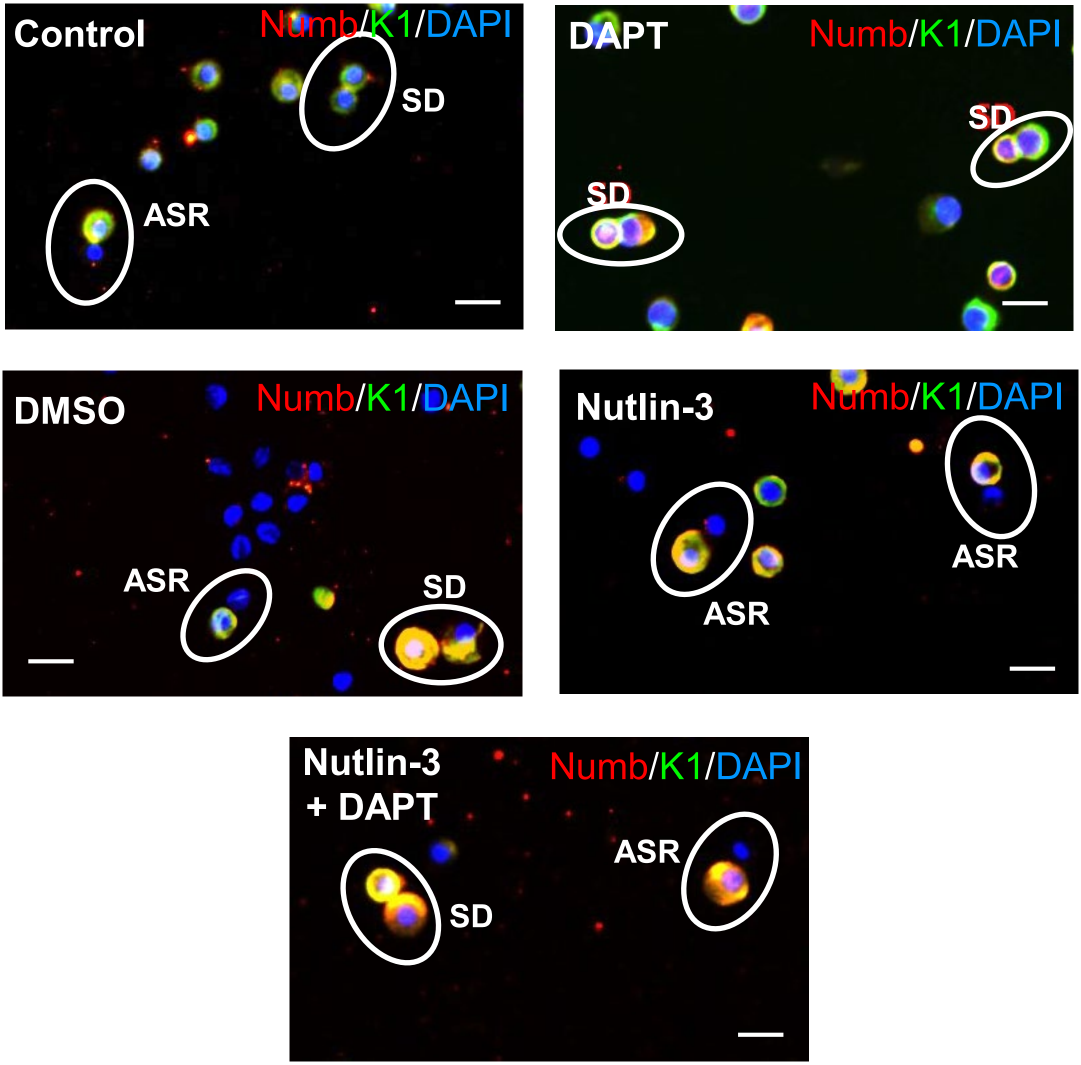

Supplementary Figure 6. Notch signaling is required for Nutlin-3-induced asymmetric SC self-renewal divisions (in support of Figure 5c). Representative asymmetric and symmetric SC self-renewal divisions and symmetric differentiation division in aged human keratinocytes under different treatment conditions. Scale bars 10μm.
